# The Therapeutic Targeting of HGF/c-Met Signaling in Hepatocellular Carcinoma: Alternative Approaches

**DOI:** 10.3390/cancers9060058

**Published:** 2017-05-26

**Authors:** Chi-Tan Hu, Jia-Ru Wu, Chuan-Chu Cheng, Wen-Sheng Wu

**Affiliations:** 1 Research Centre for Hepatology, Department of Internal Medicine, Buddhist Tzu Chi General Hospital and Tzu Chi University, Hualien 970, Taiwan; chitan.hu@msa.hinet.net; 2 Department of Laboratory Medicine and Biotechnology, College of Medicine, Tzu Chi University, Hualien 970, Taiwan; u8931246@yahoo.com.tw (J.-R.W.); cordiallove@yahoo.com.tw (C.-C.C.)

**Keywords:** hepatocellular carcinoma, metastasis, hepatocyte growth factor, c-Met, signaling transduction, therapeutic target

## Abstract

The poor prognosis of hepatocellular carcinoma (HCC), one of the most devastating cancers worldwide, is due to frequent recurrence and metastasis. Among the metastatic factors in the tumor microenvironment, hepatocyte growth factor (HGF) has been well known to play critical roles in tumor progression, including HCC. Therefore, c-Met is now regarded as the most promising therapeutic target for the treatment of HCC. However, there are still concerns about resistance and the side effects of using conventional inhibitors of c-Met, such as tyrosine kinase inhibitors. Recently, many alternative strategies of c-Met targeting have been emerging. These include targeting the downstream effectors of c-Met, such as hydrogen peroxide-inducible clone 5 (Hic-5), to block the reactive oxygen species (ROS)-mediated signaling for HCC progression. Also, inhibition of endosomal regulators, such as PKCε and GGA3, may perturb the c-Met endosomal signaling for HCC cell migration. On the other hand, many herbal antagonists of c-Met-dependent signaling, such as saponin, resveratrol, and LZ-8, were identified. Taken together, it can be anticipated that more effective and safer c-Met targeting strategies for preventing HCC progression can be established in the future.

## 1. Introduction

The poor prognosis of hepatocellular carcinoma (HCC), one of the most devastating cancers worldwide, is due to frequent recurrence and metastasis after surgical resection. In the tumor microenvironment, growth factors and cytokines, such as hepatocyte growth factor (HGF) [1,2,3,4,5] and epidermal growth factor (EGF) [6,7,8], are frequently secreted from tumor cells and/or tumor-associated stromal and inflammatory cells. Most of them are capable of triggering metastatic changes, including epithelial mesenchymal transition (EMT), enhancement of motility, and invasiveness of varieties of tumor cells [9,10,11], and thus may be collectively called “metastatic growth factors”. Among the metastatic factors, HGF has been well known to play critical roles in HCC progression. In previous clinical studies, the serum HGF level correlated positively with the tumor metastasis of HCC. Moreover, expression of c-Met, the receptor tyrosine kinase (RTK) of HGF, was closely associated with early recurrence [12]. The HGF in the HCC environment may be derived not only from tumor cells (autocrine) but also from cancer-associated cells (paracrine). Cancer cells may secrete molecules to trigger HGF expression in stromal fibroblasts, which in turn stimulate the progression of cancer cells (for review [3]). Specifically, one recent study demonstrated that HGF can be secreted from cancer-associated fibroblasts for the initiation of HCC in the context of cirrhosis [13,14]. On the other hand, in vitro studies also demonstrated the effects of HGF on phenotypical changes of HCC, including EMT, migration, and invasion [15,16,17,18]. Moreover, the c-Met receptor has been known to be a key player in drug resistance [19]. It has been established that cancer stem cells, which are capable of self-renewal and differentiation, are responsible for tumor progression and chemoresistance. Interestingly, HGF may regulate the development of cancer stem cells in HCC via c-Met/FRA1/HEY1 cascade [13,20]. Therefore, c-Met is now regarded as one of the most promising therapeutic targets for the treatment of HCC [21,22,23,24,25,26,27]. It is worth exploring the detailed mechanisms of HGF/c-Met signaling in order to identify more suitable targets to devise more effective and safer therapeutic strategies.

## 2. HGF-c-Met Signaling Mediates Tumor Progression

The receptor of HGF, c-Met, is a RTK consisting of a disulphide-linked heterodimeric complex with an extracellular portion for ligand binding, a membrane spanning segment, a juxtamembrane domain, a catalytic domain, and a C-terminal docking site [28]. Binding of the HGF to c-Met triggers dimerization and autophosphorylation of its cytoplasmic domain. Many adaptor proteins, such as Shc [29], Src, Grb2, and the p85 regulatory subunit of PI3K [28], may bind directly or indirectly to c-Met. Most of them contain a Src homologous2 (SH2) domain interacting with c-Met and a Src homologous3 (SH3) domain that binds to downstream signal molecules. Several downstream signaling pathways can be triggered by HGF/c-Met [1], including mitogen activated protein kinase MAPK) family such as ERK [30,31], p-38 [31,32], and Akt/PKB [30] pathways, which are shared by many other RTKs. HGF/c-Met can also cross talk with integrin-initiated signal cascades, leading to the activation of FAK-Src-paxillin, Ras-Rac1/Cdc42-PAK, and Gab1-Crk-C3G-Rap1 cascades [33]. Normally, the HGF-c-Met axis is critical for liver development, protection, and regeneration. However, uncontrolled HGF/c-MET signaling is one of the drivers of HCC progression [25]. HGF/c-MET signaling can be activated by metastasis associated with colon cancer 1 (MACC1) to inhibit HCC apoptosis facilitating HCC progression [34]. On the other hand, HGF-c-Met can be regulated by microRNAs, miR-26a, miR-198 [35,36], and a tumor suppressor called suppressor of cytokine signaling 1 (SOCS1) for inhibiting HCC progression [37]. Moreover, many studies demonstrated that autocrine activation of HGF/c-Met signaling was responsible for acquisition of sorafenib resistance in management of HCC [38,39].

## 3. Target Therapy Aiming at c-Met against HCC Progression

The therapeutic strategy aiming at HGF-c-Met signaling for the prevention of tumor progression of HCC was intensively investigated decades ago. Previously, many preclinical studies had strengthened the feasibility of targeting HGF-c-Met (for reviews [25,40]) for the treatment of HCC. For example, depletion of c-Met by siRNA decreased the progression of multiple HCC cell lines both in vitro and in vivo [38,41,42]. Also, PHA665752, a highly specific inhibitor of c-Met, significantly decreased the growth of subcutaneous xenografts of c-MET-overexpressing HCC cells in nude mice [43]. Currently, many clinical trials are being conducted for c-Met targeting in HCC management, using c-Met inhibitors such as INC280, foretinib, MSC2156119J, golvatinib, tivantinib, cabozantinib (for review [44]), tepotinib, and regorafenib (for review [45]). Among these, tivantinib and cabozantinib are entering phase III randomized controlled trials [44]. Also, recent clinical trials have revealed the efficacy of onartuzumab [46] and ficlatuzumab [47], antibodies against c-Met and HGF, respectively, in preventing tumor progression of solid tumors, including HCC. In addition, the potential use of c-Met-targeting miRNAs for suppressing aberrant c-Met signaling in HCC treatment is emerging [48]. In spite of this, there are still concerns about the feasibility of utilizing c-Met targeting approaches. In particular, resistance [49,50,51,52] and the side effects [53] of taking RTK inhibitors are issues that remain to be resolved.

## 4. Resistance in c-Met Targeting

There are many reports demonstrating the mechanisms for resistance to c-Met targeting, including the cross talk of c-Met with epidermal growth factor receptor EGFR [42], gene amplification of c-Met coupled with enhancement of K-RAS oncogene [39], and mutation in the activation loop of c-Met that destabilized its autoinhibitory conformation [43]. In addition, c-Met signaling may be activated in a HGF-independent fashion, such as by Des-γ-carboxy-prothrombin (DCP) [54] or cell attachment independent of the ligand [55]. Under these circumstances, inhibitors blocking the interaction of HGF and c-Met may be useless. Moreover, c-Met inhibition may trigger compensatory RTK signaling, such as EGFR/ErbB3 in HCC [56]. An additional cause of resistance is the unfeasibility of HCC patients enrolled in the trials. Previous studies indicated that c-Met overexpression was observed only in 20–48% of human HCC samples. For those HCC with negative c-Met signaling, the c-Met targeting approach will not be adequate.

## 5. Side Effects in c-Met Targeting

The toxicity of c-Met inhibitors has been demonstrated in recent studies. In animal experiments, the highly selective c-Met inhibitor GEN-203 and compound 8 may cause liver and bone marrow toxicity in mice [57] and myocardial degeneration in rats [58], respectively. In the clinical trials, severe adverse events, including leukopenia, anemia, and neutropenia, were observed in a phase 1b study of tivantinib (ARQ197) for patients with HCC [59]. In addition, foretinib caused fatigue, hypertension, and gastrointestinal toxicities for patients with papillary renal cell carcinoma [60].

The aforementioned side effects caused by c-Met inhibition may be associated with the biological functions of c-Met. HGF and c-Met are broadly expressed in epithelial cells of many organs, playing essential physiological roles [61]. Importantly, HGF/c-Met is responsible for the defensive physiological response to tissue damage and has cytoprotective activity in vivo [61]. Therefore, c-Met targeting therapy may result in the blocking of such important physiological functions, rendering the patient more susceptible to tissue damage. In addition, since many physiologically important RTKs share similar structures and functions with c-Met, they may also be affected by c-Met antagonist as off-targets.

## 6. Targeting Downstream Effector of c-Met Improves c-Met Target Therapy

There are strategies that may address the aforementioned issues in c-Met target therapy. One of them is the targeting of downstream effectors of c-Met specifically involved in tumor progression. Previously, polypeptides competing with downstream effectors of c-Met were developed. For example, antagonists of the Grb2 SH2 domain were used to prevent metastasis mediated by c-Met [62]. Moreover, indirect blocking of c-Met signaling was achieved by using specific inhibitors of downstream effectors of c-Met, such as Src, PtdIns3K/AKT, MAPK, or STAT3 [61]. However, most of the aforementioned signaling pathways are also downstream of other essential RTKs, raising the problems of unwanted targeting and unpredicted side effects. Therefore, targeting of more specific downstream effectors of c-Met for tumor progression is needed.

### 6.1. Hic-5 as a Specific Downstream Target of c-Met Pathway

One of the c-Met downstream effectors that is more feasible as a therapeutic target may be the hydrogen peroxide-inducible clone-5 (Hic-5), which was first identified in a screen for TGFβ1 and hydrogen peroxide-inducible genes [63]. Hic-5 belongs to the paxillin superfamily, the well-known focal adhesion adaptor molecules [64]. Within the paxillin superfamily, Hic-5 is the most homologous to paxillin, and both of them may play distinct, but complementary roles in triggering cancer progression [65]. In our recent report, we found that Hic-5 can be induced by HGF [66]. Moreover, Hic-5 regulated the reactive oxygen species (ROS)-JNK-signaling pathway for HCC progression induced by HGF [66]. Accordingly, we proposed that Hic-5 plays an important role in mediating c-Met signaling and tumor metastasis of HCC. One more intriguing characteristic of Hic-5 is that its tissue distribution is rather limited (normally expressed in the lung, spleen, and smooth muscle layer of tissues) as compared with paxillin (which is ubiquitously expressed), thus it may serve as a more specific and safer target for HCC therapy.

### 6.2. Targeting c-Met Endosomal Signaling of c-Met

Recently, the role of endosomal signaling in tumor progression triggered by metastatic growth factors, including HGF, has been highlighted [67]. Receptor endocytosis, either clathrin (CLA)-dependent or -independent, has a profound impact on signal transduction [68,69]. After ligand-induced RTK endocytosis, signal transduction can be sustained within the early endosome, which may recycle back to the plasma membrane or be subjected to ubiquitin-directed lysosomal degradation [68,69]. Importantly, endosomal sorting may regulate signaling pathways in a temporal and spatial manner, which is required for cell migration [70]. Moreover, HGF may recruit Golgi-localized, gamma ear-containing, Arf-binding proteins 3 (GGA3) to promote c-Met recycling and sustain ERK activation [71,72]. Several lines of evidences supported that PKC may regulate endosomal processing of RTK [68,73], including c-Met [74,75]. In our recent study, we demonstrated that HGF-induced c-Met endocytosis directs fluctuant JNK and paxillin signaling in a PKCε- and GGA3-dependent manner, leading to HCC cell migration [76]. This was achieved by c-Met degradation and recycling mediated by PKCε and GGA3, respectively [76].

Previously, c-Met endocytosis had been found to be critical in breast cancer tumorigenesis, which can be prevented by two endocytosis blockers, ConA and dynasore [77]. Recently, Kermorgant et al. suggested that the critical components within c-Met endocytic trafficking could be categorized as novel targets for the prevention of tumor progression [78]. Therefore, critical endosomal signaling components of c-Met such as PKCε- and GGA3, may be potential specific targets within c-Met signaling for preventing HCC progression.

## 7. Using Herbal Medicinal Antagonists of c-Met to Achieve More Effective and Safer c-Met Targeting

Another strategy for avoiding the problems of HGF-c-Met target therapy is the use of natural herbal drugs instead of synthetic chemical agents. For example, Timosaponin AIII (TAIII), a steroidal saponin, may suppress HGF-induced invasive activity [79]. Moreover, resveratrol, one of the major polyphenols found in red wine, prevents HCC progression by down-regulation of the HGF-c-Met signaling pathway [80]. The mechanisms for the anti-tumor effect of the herbal drugs may be rather different from those of the conventional kinase-based synthetic inhibitors. For example, Ephedrae herba, an ingredient of Maoto, which is a traditional Kampo medicine capable of preventing progression of breast cancer and mouse osteosarcoma, can downregulate the c-Met signal by promoting HGF-stimulated c-Met endocytosis and its degradation [81]. Moreover, our recent studies have demonstrated that LZ-8 (also known as Ling-Zhi or Reishi, purified from a Ganoderma lucidium) blocked both c-Met-dependent and -independent MAPK signaling for anti-HCC progression [82].

## 8. Conclusions

**Figure 1:**
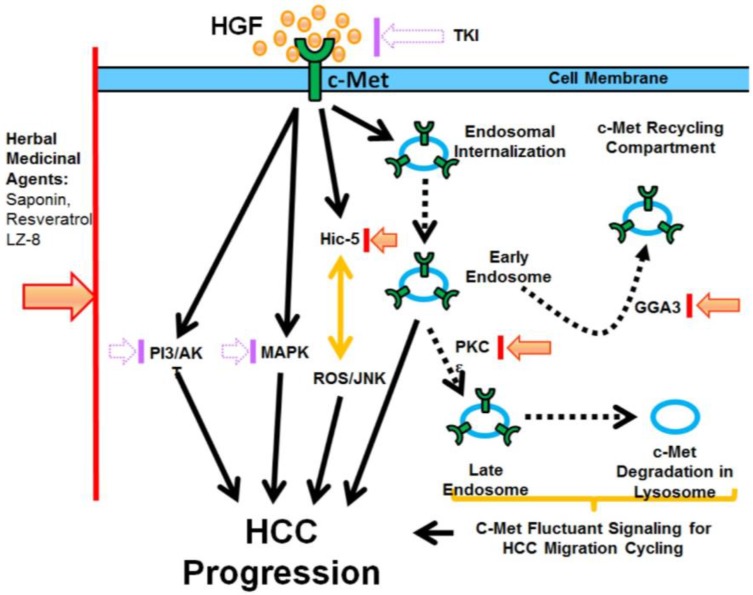
Strategies of effective targeting of hepatocyte growth factor (HGF)/c-Met signaling for the prevention of hepatocellular carcinoma (HCC). Ligand binding of c-Met triggers multiple signaling pathways, including the conventional PI3K/AKT and MAPK, coupled with the Hic-5-reactive oxygen species (ROS)-c-jun-N-terminal kinase (JNK) cascade. It also induces the c-Met endosomal pathway, including early endosomal and late endosomal, and recycling of the c-Met pathway for c-Met-mediated fluctuant signaling for HCC migration. Altogether, these pathways lead to HCC progression. Conventional blocking of c-Met signaling by a tyrosine kinase inhibitor (TKI) of c-Met or by agonists of PI3K/AKT and MAPK may cause resistance or side effects (shown as dotted arrows in purple), whereas blocking of the Hic-5-ROS-JNK cascade is proposed to be more promising (shown as solid arrows in orange-red). In addition, the inhibition of the endosomal regulators PKCε and GGA3 (shown as solid arrows in orange-red) may perturb the c-Met fluctuant signaling for cell migration. On the other hand, using herbal drugs against c-Met-dependent signaling (shown as solid arrows in orange-red) is proposed to be more effective and safer.

Target therapy aimed at the HGF/c-Met pathway is promising for the suppression of HCC progression. However, the problems of resistance and side effects in conventional kinase-based c-Met targeting remain unresolved. Many alternative strategies are emerging which aim to be more effective and safer. These include targeting the downstream effector of c-Met and using herbal drugs as safer antagonists of c-Met (summarized in Figure 1).
